# No increasing risk of a limnic eruption at Lake Kivu: Intercomparison study reveals gas concentrations close to steady state

**DOI:** 10.1371/journal.pone.0237836

**Published:** 2020-08-25

**Authors:** Fabian Bärenbold, Bertram Boehrer, Roberto Grilli, Ange Mugisha, Wolf von Tümpling, Augusta Umutoni, Martin Schmid

**Affiliations:** 1 Eawag, Swiss Federal Institute of Aquatic Science and Technology, Surface Waters—Research and Management, Kastanienbaum, Switzerland; 2 Helmholtz-Centre for Environmental Research–UFZ, Magdeburg, Germany; 3 CNRS, Université Grenoble Alpes, IRD, Grenoble INP, Institut des Géosciences de l’environnement, Grenoble, France; 4 Lake Kivu Monitoring Programme LKMP, Gisenyi, Rwanda; University of Siena, ITALY

## Abstract

Lake Kivu, East Africa, is well known for its huge reservoir of dissolved methane (CH_4_) and carbon dioxide (CO_2_) in the stratified deep waters (below 250 m). The methane concentrations of up to ~ 20 mmol/l are sufficiently high for commercial gas extraction and power production. In view of the projected extraction capacity of up to several hundred MW in the next decades, reliable and accurate gas measurement techniques are required to closely monitor the evolution of gas concentrations. For this purpose, an intercomparison campaign for dissolved gas measurements was planned and conducted in March 2018. The applied measurement techniques included on-site mass spectrometry of continuously pumped sample water, gas chromatography of in-situ filled gas bags, an in-situ membrane inlet laser spectrometer sensor and a prototype sensor for total dissolved gas pressure (TDGP). We present the results of three datasets for CH_4_, two for CO_2_ and one for TDGP. The resulting methane profiles show a good agreement within a range of around 5–10% in the deep water. We also observe that TDGP measurements in the deep waters are systematically around 5 to 10% lower than TDGP computed from gas concentrations. Part of this difference may be attributed to the non-trivial conversion of concentration to partial pressure in gas-rich Lake Kivu. When comparing our data to past measurements, we cannot verify the previously suggested increase in methane concentrations since 1974. We therefore conclude that the methane and carbon dioxide concentrations in Lake Kivu are currently close to a steady state.

## Introduction

Lake Kivu, with a surface area of 2386 km^2^ and a maximum depth of 485 m, is situated on the border between Rwanda and the Democratic Republic of the Congo (DRC). Along with other African great lakes Tanganyika and Malawi, Lake Kivu is part of the East African Rift System (EARS). To the north, Lake Kivu borders on the Virunga volcano chain, while to the South it drains into Lake Tanganyika via the Ruzizi River. Lake Kivu is fed by numerous small streams [1] and by subaquatic groundwater sources [2] with the latter contributing about 45% of the total inflow. The groundwater sources mainly enter the lake at the northern shore and can be divided into two categories: two cool and fresh sources above a depth of 260 m and several warm, saline and carbon dioxide (CO_2_)-rich sources below 260 m. This has two main consequences, namely a very stable density stratification due to the salinity gradient, which prevents annual mixing below a depth of 50 to 60 m and the accumulation of dissolved CO_2_ over long time scales. In addition to CO_2_, biogenic methane (CH_4_) is present in the deep waters in large amounts due to decomposition of organic matter at the lake bottom and CO_2_ reduction [3, 4].

Gas concentrations in Lake Kivu were first recorded by Damas in 1935 [5] who measured CO_2_ and H_2_S. However, Damas only analyzed the sample water after degassing, thus losing more than half of the CO_2_ to the atmosphere. Between 1952 and 1954, Schmitz and Kufferath carried out the first CH_4_ measurements and additionally determined CO_2_ concentrations. However, they only analyzed the gas that outgassed under atmospheric conditions, neglecting the gas remaining dissolved in the water [6]. In 1974, Tietze performed the first comprehensive survey of dissolved gas concentrations, including CH_4_ and CO_2_ from both the gas exsolved under atmospheric conditions and the remaining part in the sample water [7]. Tietze concluded that about 300 km^3^ STP (gas volume at 0°C and 1 atm) of CO_2_ and 60 km^3^ STP of CH_4_ were stored in the permanently stratified deep waters (below ~ 60 m) of Lake Kivu [7]. Subsequently, based on new measurements from M. Halbwachs and J.-C. Tochon in 2003 (published in [8]), Schmid et al., 2005 suggested that CH_4_ concentrations had increased by 15% since 1974 and that they could possibly reach saturation within the 21^st^ century. With the examples of deadly limnic eruptions due to high gas loads in Lakes Nyos [9] and Monoun [10], it was clear that the gas concentrations of Lake Kivu needed to be monitored. Besides the threat to the local population, the gas content in Lake Kivu also represents a valuable resource: In December 2015, a 26 MW gas power plant was connected to the Rwandan grid and several hundred MW could follow according to projections [11].

In 2017, a gas intercomparison campaign was initiated by the Lake Kivu Monitoring Programme (LKMP) with the goal of 1) accurately determining CH_4_ and CO_2_ concentrations using different methodologies and 2) finding an appropriate technique to regularly monitor the gas concentrations in the future. However, gas sampling in highly outgassing environments is challenging and thus, the measurement methods had to be adapted accordingly. In this work, we describe the methodologies for three research teams involved in the campaign: The Swiss Federal Institute of Aquatic Science and Technology (Eawag), the Helmholtz Centre for Environmental Research (UFZ) and the National Center of Scientific Research in France (CNRS). Subsequently, we present the results of each group and compare them to the previous measurements of Tietze in 1974 [7] and Halbwachs and Tochon in 2003 and Schmid in 2004 (both published in [8]). Finally, we reevaluate the gas (CH_4_ and CO_2_) content in Lake Kivu and its potential change in time.

## Materials and methods

The intercomparison campaign took place close to Gisenyi/Rubavu, Rwanda (1.74087°S / 29.22602°E) from 9 to 13 March 2018 and involved research teams from Eawag, UFZ, CNRS and from the power plant operator KivuWatt. Eawag prolonged its measurement period until 18 March and UFZ also included earlier measurements from 2017. The campaign was planned and organized by LKMP and therefore, no special permit was necessary to perform measurements on the lake. Further details on the results of the campaign can also be found in a report to LKMP [12]. Note that while the report includes the measurements of KivuWatt, the latter decided to not be part of this publication.

The measurements taken by each research team are summarized in Table 1. In this publication, only the approach of Eawag is explained more comprehensively, while further details on the methods of UFZ and CNRS can be found elsewhere ([13, 14]). In the following, we will first present the methodology of Eawag and then shortly summarize the approaches of UFZ and CNRS.

**Table 1:** Summary of gas measurements performed by the different research teams of Eawag, UFZ and CNRS.

	CH_4_ 0–150 m	CH_4_ 150–450 m	CO_2_	TDGP
Eawag	**-**	**+**	**+**	**-**
UFZ	**-**	**+**	**+**	**+**
CNRS	**+**	**-**	**-**	**-**

The “+” indicates which measurements were performed by which groups.

### Measurement method used by Eawag

**Fig 1:**
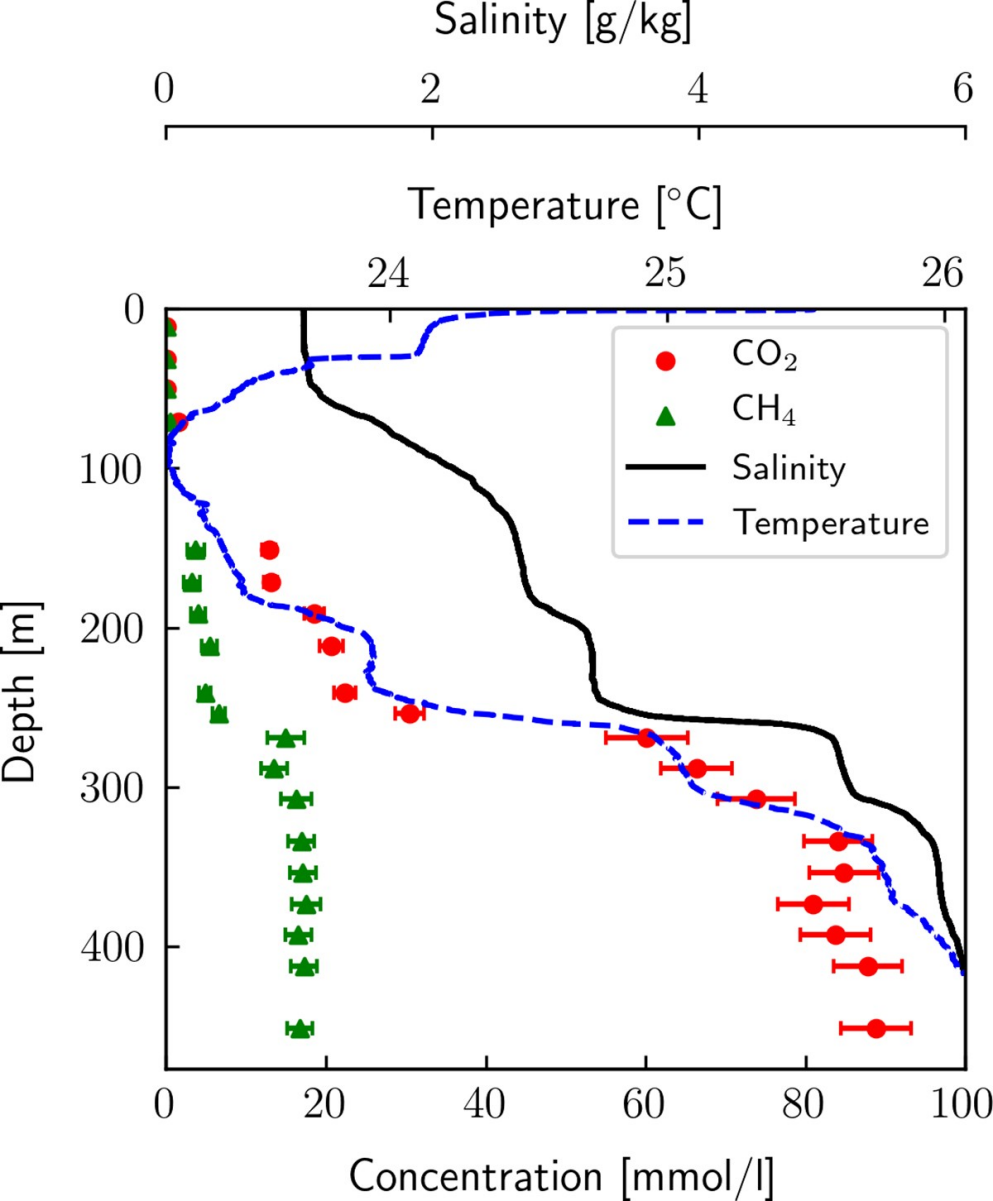
Dissolved CH_4_ and CO_2_ profiles by Eawag. Dissolved CH_4_ and CO_2_ concentrations measured in Lake Kivu by Eawag in 2018; salinity and temperature were determined using a CTD from Sea and Sun in 2017 and 2018 respectively. The conversion from conductivity to salinity was done according to [28] using ionic composition. Gas concentrations between 90 and 130 m could not be measured due to too high gas load for using a membrane contactor only, but not high enough for the use of our equilibration chamber.

The measurement setup was built around “miniRuedi”, a gas-equilibrium portable membrane-inlet mass spectrometric system (GE-MIMS) which allows on-site quantification of different dissolved gases in water (i.e. N_2_, O_2_, CO_2_, CH_4_, He, Ar, see [15]). The continuous sampling water flow (~ 1 L/min) required to maintain gas equilibrium at the MS inlet was provided by a submersible pump (0.75 kW, Lechner Pumpen) and 250 m long, 6 mm inner diameter polyamide (PA) tubing. The pump was used only above 250 m and yielded a flow of ~ 1.6 L/min. Below 250 m, TDGP increases drastically and, following initiation of the flow by a suction pump at the surface, the buoyancy due to bubble formation within the tube was sufficient to lift the water to the surface. 10 mm PA tubing was used in this case and the resulting flow was ~ 1 L/min (except between 270 and 310 m where it was ~ 0.5 L/min only). The water-gas mixture was subsequently dispersed through a nozzle into a custom-made cylindrical equilibration chamber (12.3 cm diameter, 38 cm height, see Fig 1 in S1 Appendix). While the degassed sample water accumulated and discharged at the bottom of the chamber, the gas phase stayed above and left the chamber through a tube at the top. The gas content in the gas phase and the water phase (via a headspace created by the membrane contactor Liqui-Cel G542, 260 cm^3^ external volume) was analyzed by the “miniRuedi”. Finally, gas and water flow rates were recorded to compute in-situ gas concentrations in the lake as sketched in Fig 1 in S1 Appendix. The overall analytical accuracy (i.e. the maximum deviation from the true value) of the setup was deduced from the accuracies of its individual components and estimated to around ± 5% for CO_2_ and ± 10% for CH_4_ in the deep water (see S1 Appendix for more details). Measurements were done at a resolution of 20 m starting from 10 m depth down to 450 m (430 m was omitted due to time constraints). Between 90 and 130 m, the gas flow was too low to be quantified but still substantial enough to have an effect on gas results. Therefore, results for this depth range are not reported.

The mass spectrometer was calibrated using two gas standards (80% CO_2_ + 20% CH_4_ and 60% CO_2_ + 30% CH_4_ + 10% air) with partial pressures similar to the average gas composition of water gassing out from Lake Kivu deep water. One of the gas standards additionally contained atmospheric air for potential calibration of N_2_ and O_2_. However, in the special setting of Lake Kivu, the determination of N_2_ at mass/charge = 28 proved to be difficult because of the presence of a large peak of CO from the fragmentation of CO_2_ during ionization in the mass spectrometer. The interference of the CO fragment accounted for more than 95% of the intensity at mass 28. Therefore N_2_ could not be determined reliably and hence was not included in this publication.

### Measurement method used by UFZ

The measurement method used by UFZ had previously been used in highly gas charged mine pit lakes (for CO_2_ see [16], for CH_4_ see [17]) and was modified for the conditions of Lake Kivu by [13]. Water was sampled using gas-tight bags, which were lowered to the appropriate depth together with a small pump and an automatic pump controller. The pump partially filled the bags while leaving enough space for the gas phase, which forms once the bags are retrieved. At the surface, the water and gas phases in the bags were equilibrated over night and the composition of the gas phase was analyzed using a gas chromatograph. Subsequently, the remaining amount of gas in the water phase was deduced by assuming equilibrium between gas and water phase. In order to compute in-situ gas concentrations, the gas and water volumes in the bag were determined using a syringe and a laboratory scale respectively. Total uncertainties for CH_4_ (CO_2_) concentrations were determined as ± 5 (± 6) % below and ± 7 (± 8) % above 250 m. Note again that these uncertainties should be interpreted as maximum deviation from the true value. The UFZ group also measured total dissolved gas pressure (TDGP) using a prototype probe from Pro Oceanus with an accuracy of ± 0.04 bar according to the manufacturer.

### Measurement method used by CNRS

The measurement method applied by CNRS is fully described elsewhere [14]. In short, an in-situ membrane-inlet laser spectrometer (MILS), called SubOcean, was deployed for continuous dissolved CH_4_ measurements. The instrument is based on a patented membrane extraction system [18] coupled to an optical spectrometer for trace gas sensing based on an optical feedback cavity enhanced absorption spectroscopy (OFCEAS) technique [19, 20]. The extraction system does not rely on gas equilibration across the membrane, but the dry side of the membrane is maintained at low pressure while continuously flushing it with dry zero air. This allows achieving fast response times < 30 sec, making the technique adapted for fast 3D mapping of water masses [21]. The accuracy (standard deviation at the three sigma level) of the measurements was quantified to be ± 33% from repeated measurements at the same depth. Note that the uncertainty is given at the three sigma level, which we judge roughly equivalent to the concept of “maximum deviation from the true value” used for the other two methods. In addition to the CH_4_ signal, this method needs external TDGP and dissolved CO_2_ measurements in order to compute CH_4_ partial pressure in the lake water, which is finally converted to concentrations using the conversion method presented below.

### Conversion from concentration to partial pressure

Eawag and UFZ measured gas concentrations while the MILS sensor used by CNRS provided partial gas pressure. In order to compare these results, conversion from concentration to partial pressure and vice-versa is required. However, this conversion is not straightforward as the gas-water partition coefficients (Henry coefficients) depend on temperature, salinity and hydrostatic pressure [22]. In addition, the fugacity effect cannot be calculated separately for each gas since it depends on the relative mixture of the involved gas species [23]. We thus express concentration *C*_*i*_ as a function of partial pressure *p*_*i*_ by the following equation:

$$C_{i}={K_{i}(T,S,P)p}_{i}\varphi_{i}(T,P)$$ (1)

with *φ*_*i*_ the fugacity coefficient i.e. the ratio between the fugacity of a gas and its partial pressure at temperature T and pressure P, and *K*_*i*_ the solubility coefficient, i.e. the ratio between the dissolved concentration of a gas and its fugacity. The solubility coefficient *K_i_*(*T,S*) is computed as a function of temperature and salinity according to [22] for CO_2_, [24] for CH_4_ and [25] for N_2_. The salinity terms of these equations were originally derived for sea salt and not for Lake Kivu, where salinity is dominated by bicarbonates. We accounted for this by assuming that the salinity effect mainly depends on the ionic strength of the solution. More details about the salinity correction are provided in S2 Appendix.

According to [22], the dependence of the solubility coefficient *K*_*i*_ on local total pressure (hydrostatic plus atmospheric pressure) can be written as

$$K_{i}(T,S,P)=K_{i}(T,S)e^{\left[\frac{(1-P)v_{i}}{RT}\right]}.$$ (2)

Here, R = 83.1446 cm^3^ bar K^−1^ mol^−1^ is the gas constant, and *v*_*i*_ are the partial molar volumes (cm^3^ mol^-1^). The partial molar volumes of CO_2_ and N_2_ were assumed constant at 32.3 cm^3^ mol^-1^ [22] and 35.7 cm^3^ mol^-1^ [26] respectively, while for CH_4_ it was calculated according to [27].

The resulting pressure correction factors *K_i_*(*T,S,P*)/*K_i_*(*T,S*) range between 1.00 at atmospheric pressure and 0.93 (CO_2_) or 0.94 (CH_4_) at 50 bar (485 m depth), i.e. the in-situ pressure reduces the solubility coefficient of the gases by 6 to 7% in the lowest layers of Lake Kivu.

The fugacity coefficients *φ_i_*(*T,P*) of CO_2_, CH_4_ and N_2_, including the interaction between the gases, are computed according to [23] (Octave scripts available in S3 Appendix).

## Results and discussion

### Eawag results

The resulting CH_4_ and CO_2_ concentrations using the Eawag mass spectrometer setup are depicted in Fig 1, along with temperature and salinity profiles. We can identify the well-mixed epilimnion with constant salinity (above ~ 60 m) and the main chemocline at ~ 255 m. As expected, gas concentrations correlate well with salinity and temperature due to the common hydrothermal origin of CO_2_, dissolved solids and heat [8]. The detailed CH_4_ and CO_2_ results can be found in S1 Table.

Contamination with atmospheric air is a major source of measurement errors in gas content analysis. Thus, we use our O_2_ results to estimate the maximum atmospheric contamination affecting our measurements in the completely anoxic deep waters of Lake Kivu. We find that the mixing ratio of O_2_ in the sampled gas phase is always less than 1% (maximum between 270 and 310 m due to lower water flow at these depths). Most likely, this O_2_ signal indicates a small contamination with atmospheric air. However, it could stem from gas fragmentation during ionization in the mass spectrometer. Independently of its origin, the signal is small enough to not significantly affect the CH_4_ and CO_2_ results.

### Intercomparison of CH_4_ and CO_2_ using past and new measurements

**Fig 2:**
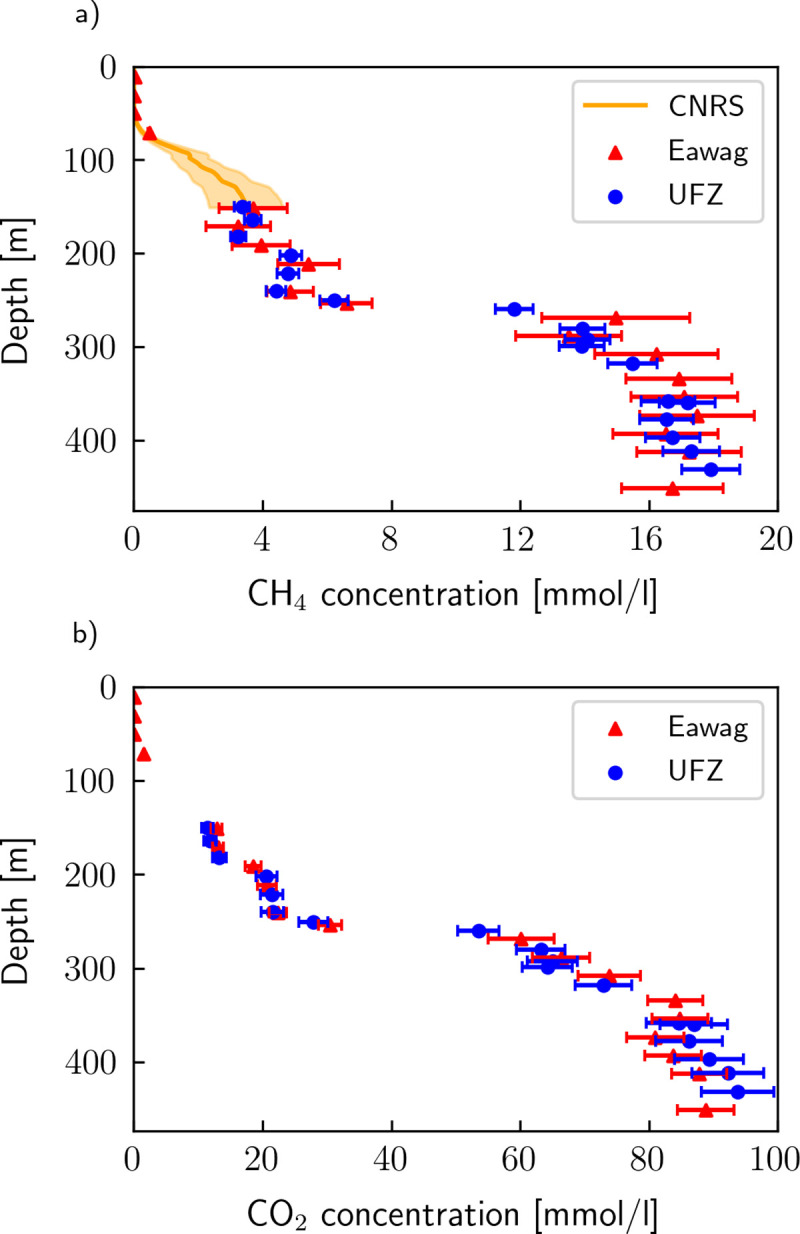
Comparison of CH_4_ and CO_2_ results from CNRS, Eawag and UFZ in 2018. Eawag and UFZ directly determined gas concentration while CNRS measured quasi-continuous partial CH_4_ pressure, which was converted to concentration using the conversion method presented above.

The results of the dissolved gas measurements (CH_4_ and CO_2_) of Eawag, UFZ [13] and CNRS [29] are shown in Fig 2. For both CH_4_ and CO_2_, the measurements agree well within the uncertainties of the different approaches. The profile from Eawag shows higher CH_4_ concentrations (up to 10%) than UFZ between 250 and 350 m depth, whereas UFZ measured higher CH_4_ and CO_2_ concentrations (up to 5%) below 400 m. In particular, the UFZ profile indicates further increasing CH_4_ and CO_2_ concentrations with depth below 400 m, while the Eawag profile levels off or even decreases. However, note that the comparison below 400 m is based on very few measurement points. The results of Eawag, UFZ and CNRS show a good agreement at their junction at 150 m, thus validating the conversion method under moderate hydrostatic pressure.

In order to estimate the total gas content in the lake, we need to derive quasi-continuous depth profiles for CH_4_ and CO_2_ from the measurements depicted in Fig 2. We chose to interpolate the discrete profiles by fitting them to an electric conductivity profile (corrected to 25°C), because i) conductivity is most probably closely related to gas concentrations due to the long residence time in the lake and because similar transport processes affect both dissolved solids and gases [30] and ii) it can be easily measured at a high resolution.

**Fig 3:**
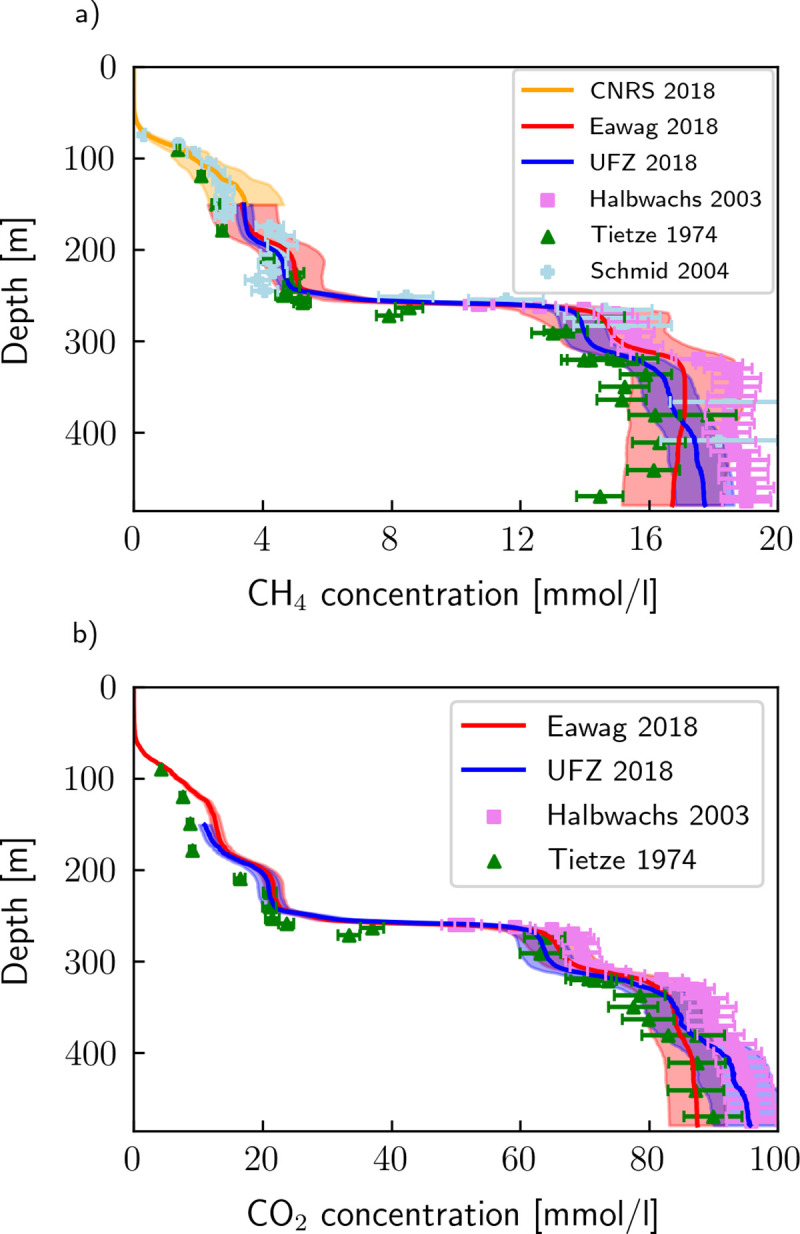
Interpolated CH_4_ and CO_2_ concentrations from this work compared to previous measurements. CH_4_ and CO_2_ concentrations of Eawag and UFZ are interpolated by fitting the profiles to a conductivity profile; shaded areas represent the uncertainties. Selected previous gas measurements are shown for comparison [7, 8].

The following procedure was applied to derive high-resolution curves for the CH_4_ and CO_2_ concentrations measured by UFZ and Eawag: The conductivity profile from Fig 1 was extended down to 480 m depth using the background conductivity profile published by [2]. The latter was corrected with the mean difference between both profiles in their lowest common 20 m. From this profile, we then extracted the conductivity values at the depths of the gas measurements of UFZ and Eawag. Then, a 6^th^ order polynomial function was fitted (R^2^ > 0.995 for all four profiles) with conductivity as the independent and gas concentrations as the dependent variables. The regression was used to compute the gas concentration as a function of conductivity and to relate it to depth. The resulting curves are shown in Fig 3 (at 0.5 m resolution), along with previous CH_4_ and CO_2_ measurements. The uncertainties of the previous measurements were assumed to be ± 5% for Tietze [7], ± 4% for Halbwachs and Tochon [8] and ± 10% for Schmid ([8] and pers. comm. M. Schmid).

In general, previous and current measurements are in good agreement. For both CH_4_ and CO_2_, the measurements of 1974 are at the lower end of the spectrum, and those of 2003 at the higher end. From this fact, [8] concluded that CH_4_ concentrations had increased by 15% from 1974 to 2003 and that they could possibly reach saturation within the 21^st^ century. Pasche et al. [4] later determined an upper bound for the CH_4_ increase of around half this value based on carbon cycle analysis. However, our new measurements show no measurable increase of CH_4_ (and CO_2_) concentrations within the last 45 years. This implies that the measured differences were largely due to measurement uncertainty and that the CH_4_ and CO_2_ concentrations in the lake are currently close to a steady state.

### Risk assessment using total dissolved gas pressure (TDGP)

In order to assess the danger of a potential gas eruption associated with the high gas concentrations in Lake Kivu, it is helpful to look at gas pressure saturation within the lake. CH_4_ and CO_2_ concentrations thus need to be converted to partial pressure using the conversion method presented in the previous section. Besides CH_4_ and CO_2_, dissolved nitrogen (N_2_) is the only gas present in sufficient amounts to influence gas pressure. As no N_2_ data is available for Lake Kivu, we estimated its contribution assuming that it mimics the profile of atmospheric noble gases which show concentrations close to air saturated water (ASW) at the lake surface and a decrease of ~50% in the deep water [31]. The derived N_2_ profile was subsequently included in the conversion algorithm, which includes the effect of gas mixture between CH_4_, CO_2_ and N_2_ on the fugacity coefficients.

The accuracy of the calculated TDGP is estimated from the accuracies of the gas/water flow measurements and the CH_4_ concentration in the gas phase. The contributions to the accuracy from CO_2_ and N_2_ are negligible in comparison to CH_4_ because of the high solubility of CO_2_ and low concentration of N_2_.

**Fig 4:**
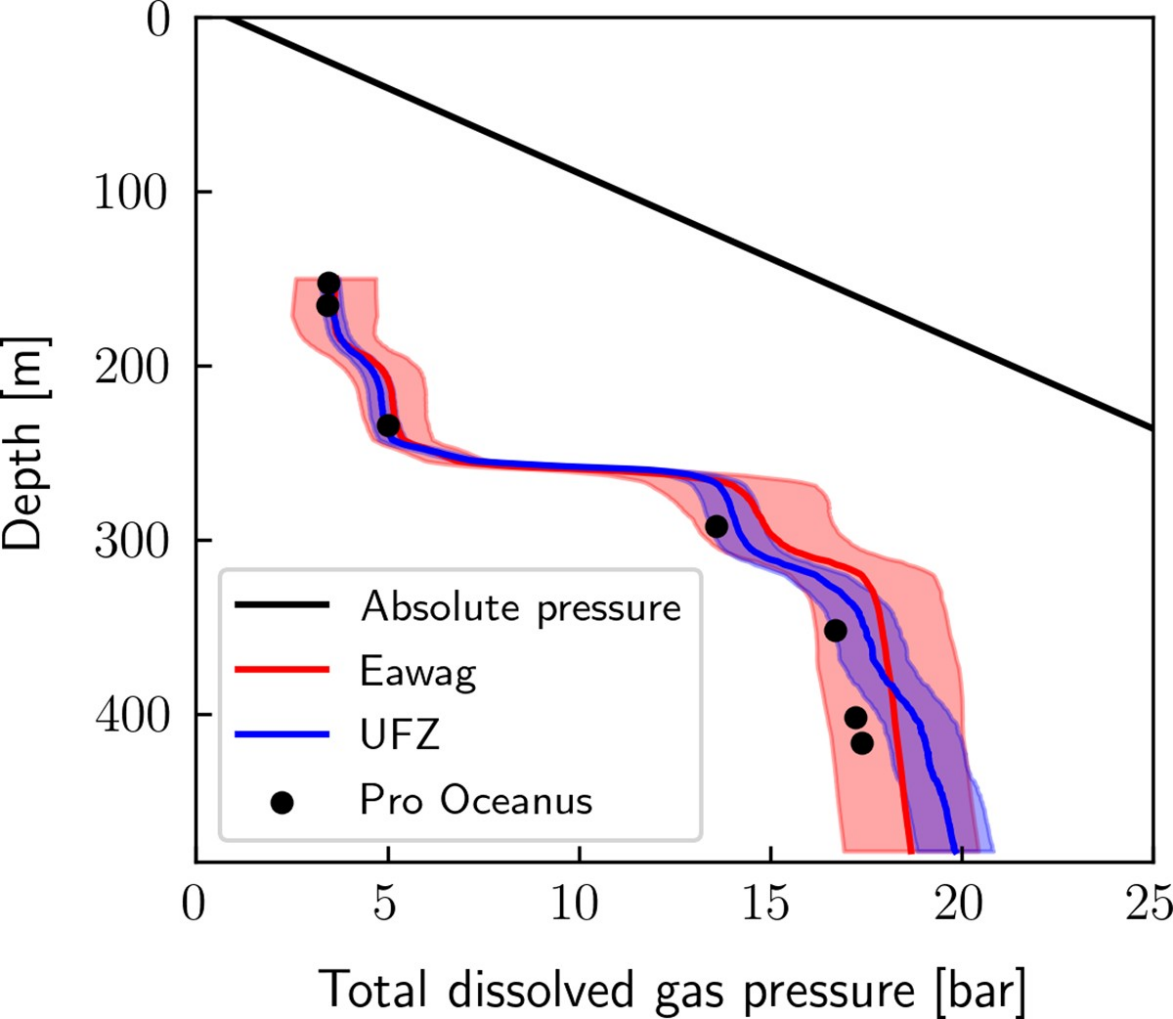
Comparison of measured TDGP with TDGP calculated from gas concentrations. TDGP is calculated from individual gas components (CH_4_, CO_2_ and N_2_) using the conversion algorithm presented in this work. The concentration profile of N_2_ was derived assuming a similar depth profile as Neon [**31**] thus not accounting for possible biological effects on N_2_.

Fig 4 shows that calculated TDGP of Eawag and UFZ and direct TDGP measurements using the Pro Oceanus sensor are in good agreement, usually well within the uncertainties of the respective methods. Still, below 250m depth, TDGP calculated from Eawag and UFZ data is slightly higher than the measured TDGP. The mean difference between calculated and measured TDGP is 5.9% (maximum of 8.7% at 290 m) and 4.4% (maximum of 8.8% at 417 m) for Eawag and UFZ respectively. This discrepancy could be due to a bias in the conversion of concentrations to partial pressures, to an overestimation of concentrations by both Eawag and UFZ or to a problem of calibration of the TDGP sensor at high pressure.

Gas concentrations are very high in the deep water of Lake Kivu. If this gas was released to the atmosphere, it would cause a large catastrophe by suffocating humans and animals in the surrounding area, qualitatively similar to the events at Lake Nyos in 1986 [9]. Currently, total dissolved gas pressure (TDGP) is well below absolute pressure (hydrostatic plus atmospheric pressure) at all depths in Lake Kivu. The maximum gas saturation in terms of pressure is reached at 320 m and amounts to ~ 50% (or a maximum of 57% if we take the upper limit of the uncertainty range of the Eawag data). This means that the gas concentrations are still far away from the point of spontaneous ebullition (i.e. close to 100% saturation). Nevertheless, volcanic structures on the lake floor indicate frequent volcanic activity within the lake in the geologically recent past [32]. We cannot exclude that similar volcanic activity could trigger a gas eruption from the lake in the future, even though TDGP is far away from saturation. Therefore, in spite of no measurable increase of gas concentrations during the last 45 years, artificial degassing is still beneficial to reduce the danger of a potential natural disaster.

### Update of Lake Kivu gas reserves

We estimated the gas content of Lake Kivu by multiplying our interpolated gas profiles (Fig 3) by the lake area at each depth and subsequent integration over the lake depth at a resolution of 0.5 m. The lake areas were deduced from the bathymetry of Lake Kivu by K.A. Ross from the blended bathymethric data of [32, 33]. Tables 2 and 3 show the gas content in different depth ranges for CH_4_ and CO_2_ respectively. The average CH_4_ estimate from the 2018 campaign shows slightly lower values in the resource zone (7%) and in the entire lake (4.5%) than calculated from the data of Halbwachs and Tochon 2003 (published in [8]). Similarly, the CO_2_ content measured in 2018 is 6.5% lower in the resource zone and 3% lower for the entire lake. We do not think that this apparent decrease in gas concentrations since 2003 reflects the real gas dynamics in Lake Kivu because i) the total CH_4_ extracted by the existing power plant was less than 0.2 km^3^ until March 2018 [11] and therefore not measurable by current methods, ii) the residence time of gases in the deep water is on the order of 1000 years [34] and iii) to our knowledge, there is no process that would consume either CO_2_ or CH_4_ under the conditions present in the deep water of Lake Kivu (i.e. below 70 m [35]).

**Table 2:** CH_4_ content in Lake Kivu in km^3^ STP for different depth ranges.

Depth range [m]	Eawag 2018	UFZ 2018	CNRS 2018	Average	Halbwachs 2003
0–70	-	-	0.1 ± 0.3	0.1	
70–150	-	-	6.5 ± 2.1	6.5	
150–200	5.8 ± 1.6	5.7 ± 0.4	-	5.7	
200–260	8.5 ± 1.3	8.2 ± 0.5	-	8.3	8.5
260–300	12.5 ± 1.7	12.1 ± 0.6	-	12.3	
300–350	14.7 ± 1.6	13.8 ± 0.7	-	14.2	
350–400	9.5 ± 0.9	9.4 ± 0.5	-	9.5	
400–480	5.5 ± 0.5	5.7 ± 0.3	-	5.6	
Resource zone (260–480)	42.2 ± 4.8	40.9 ± 2.0	-	41.5	44.7
Upper resource zone (260–310)	15.6 ± 2.1	15.0 ± 0.7	-	15.3	
Lower resource zone (310–480)	26.6 ± 2.6	26.0 ± 1.3	-	26.3	
Entire lake				62.2	65.1

The reference values (Halbwachs 2003) were calculated in [34] based on the data of M. Halbwachs and J.-C. Tochon in [8]. The resource zones are defined as in [11], but including half of the bordering gradients.

**Table 3:** CO_2_ content in Lake Kivu in km^3^ STP for different depth ranges.

Depth range [m]	Eawag 2018	UFZ 2018	Average	Halbwachs 2003
0–150	24.7 ± 2.1	-	24.7	
150–200	23.4 ± 1.5	21.7 ± 1.5	22.5	
200–260	37.0 ± 2.3	37.0 ± 2.4	37.0	38
260–300	56.2 ± 4.2	55.2 ± 2.8	55.7	
300–350	68.7 ± 4.0	66.8 ± 3.4	67.7	
350–400	47.2 ± 2.4	48.2 ± 2.4	47.7	
400–480	28.1 ± 1.4	30.1 ± 1.5	29.1	
Resource zone (260–480)	200.2 ± 12.1	200.3 ± 10.0	200.2	214
Upper resource zone (260–310)	69.9 ± 5.1	68.2 ± 3.4	69.1	
Lower resource zone (310–480)	130.3 ± 6.9	132.1 ± 6.6	131.2	
Entire lake	285.3 ± 18.0		284.5	294

The reference values (Halbwachs 2003) were calculated in [34] based on the data of M. Halbwachs and J.-C. Tochon in [8]. The resource zones are defined as in [11], but including half of the bordering gradients.

Schmid et al. [8] suggested a CH_4_ production rate of 120 g C/m^2^/year (grams of carbon in CH_4_ per sediment area pear year) in order to explain the difference between the CH_4_ profiles of K. Tietze in 1974 and M. Halbwachs and J.-C. Tochon in 2003. This rate would lead to a CH_4_ increase of about 5–10% since 2003 (i.e. 3–6 km^3^) and thus can be excluded based on our data. Similarly, we can rule out a production rate of 93 g C/m^2^/year in the deep water as proposed by [4]. Based on our data from 2018, we suggest that the actual production rate of CH_4_ is probably close to the steady state rates of 32 and 35 g C/m^2^/year calculated by [4, 8] respectively.

We conclude that the variability of gas concentrations measured in the last 45 years is due to the uncertainties of the applied methods. In contrast to previous work [8], our data suggests that the lake gas content is currently close to a steady state with no or small net recharge rate. Consequently, the risk of a gas eruption does not seem to be increasing over time. Additionally, our findings question whether the methane in Lake Kivu is replenished fast enough to be used as a long-term energy source, once the current methane storage has been exploited.

The CH_4_ content amounts to around 41.5 km^3^ STP in the resource zone (between 260 and 480 m) and 62.2 km^3^ in the whole lake. Furthermore, the results of the two methodologies suitable for deep water gas analysis (Eawag and UFZ) agree within the expected accuracy of 5–10% for both CH_4_ and CO_2_. For regular gas monitoring in view of increased industrial gas extraction, the method of UFZ is easier to apply due to the use of relatively simple equipment. The prototype MILS sensor used by CNRS was able to record gas concentrations down to a depth of 150 m and provides the advantage of in-situ, fast and high-resolution data. However, the technique was not yet adapted to the high CH_4_ concentration below 150 m in Lake Kivu. In addition, the sensor requires total dissolved gas pressure (TDGP) and dissolved CO_2_ profiles to determine CH_4_ partial pressure. The direct measurement of TDGP may be the most appropriate measurable quantity to monitor the risk of a spontaneous ebullition in the lake in the future. It also has the advantage of offering simple, reproducible and high-precision measurements for further monitoring purposes (see for example [36]).

## Supporting information

S1 Table Detailed results of Eawag measurement method. (DOCX)

S2 Table Temperature data from a Sea and Sun CTD. (TXT)

S3 Table Salinity data computed using conductivity data. (TXT)

S1 Appendix Detailed description of Eawag measurement method and calculations. (DOCX)

S2 Appendix Calculation of salinity effect of Lake Kivu dissolved solids. (DOCX)

S3 Appendix Octave scripts for conversion of concentration to partial pressure. (DOCX)
