# Supplementary material for: No increasing risk of a limnic eruption at Lake Kivu: Intercomparison study reveals gas concentrations close to steady state
Source: PLoS One. 2020 Aug 25;15(8):e0237836. doi: 10.1371/journal.pone.0237836 (PMC7446963; doi:10.1371/journal.pone.0237836)
Supplement: S1 Table — (DOCX) [file pone.0237836.s001.docx]

**No increasing risk of a limnic eruption at Lake Kivu: intercomparison study reveals gas concentrations close to steady state**

Fabian Bärenbold^1^*, Bertram Boehrer^2^, Roberto Grilli^3^, Ange Mugisha^4^, Wolf von Tümpling^2^, Augusta Umutoni^4^, Martin Schmid^1^

**S1 Table. Detailed results of Eawag measurement method.** Results and uncertainties using the on-site mass spectrometer method

| **Depth [m]** | **CH_4_ [mmol/l]** | **CH_4_ err [mmol/l]** | **CO_2_ [mmol/l]** | **CO_2_ err [mmol/l]** |
| --- | --- | --- | --- | --- |
| 11 | <0.1 |  | 0.03 | 0.00 |
| 32 | <0.1 |  | 0.05 | 0.00 |
| 50 | <0.1 |  | 0.05 | 0.00 |
| 71 | 0.47 | 0.03 | 1.54 | 0.07 |
| 91 |  |  | 5.08 | 0.22 |
| 112 |  |  | 7.57 | 0.33 |
| 132 |  |  | 9.30 | 0.41 |
| 152 | 3.70 | 1.06 | 12.86 | 0.81 |
| 172 | 3.24 | 1.00 | 13.07 | 0.85 |
| 192 | 3.96 | 0.90 | 18.50 | 1.22 |
| 212 | 5.42 | 0.96 | 20.64 | 1.48 |
| 242 | 4.85 | 0.71 | 22.34 | 1.33 |
| 254 | 6.60 | 0.79 | 30.48 | 1.77 |
| 270 | 14.97 | 2.29 | 60.13 | 5.15 |
| 289 | 13.50 | 1.65 | 66.37 | 4.44 |
| 309 | 16.23 | 1.92 | 73.86 | 4.83 |
| 335 | 16.93 | 1.64 | 84.05 | 4.30 |
| 355 | 17.09 | 1.66 | 84.79 | 4.33 |
| 375 | 17.49 | 1.78 | 80.94 | 4.43 |
| 394 | 16.51 | 1.63 | 83.72 | 4.41 |
| 414 | 17.24 | 1.62 | 87.78 | 4.31 |
| 453 | 16.73 | 1.57 | 88.82 | 4.36 |
