# Supplementary material for: No increasing risk of a limnic eruption at Lake Kivu: Intercomparison study reveals gas concentrations close to steady state
Source: PLoS One. 2020 Aug 25;15(8):e0237836. doi: 10.1371/journal.pone.0237836 (PMC7446963; doi:10.1371/journal.pone.0237836)
Supplement: S1 Appendix — (DOCX) [file pone.0237836.s004.docx]

**No increasing risk of a limnic eruption at Lake Kivu: intercomparison study reveals gas concentrations close to steady state**

Fabian Bärenbold^1^*, Bertram Boehrer^2^, Roberto Grilli^3^, Ange Mugisha^4^, Wolf von Tümpling^2^, Augusta Umutoni^4^, Martin Schmid^1^

**S1 Appendix. Detailed description of on-site measurement method for CO_2_ and CH_4_**

Overview

The measurement approach is schematically summarized in Figure 1. Lake water is pumped to the surface continuously using a 0.75 kW submersible centrifugal pump (UG-18 from Pumpen Lechner) down to a depth of 250 m. Below 250 m, no pump is needed because the outgassing of deep water in the tube sustains the water flow. However, a small peristaltic pump is used to initiate the process by bringing deep water close to the lake surface where the hydrostatic pressure is too low to keep the gases in the water. Relevant data about the pump operation above/below 250 m are shown in Table 1.


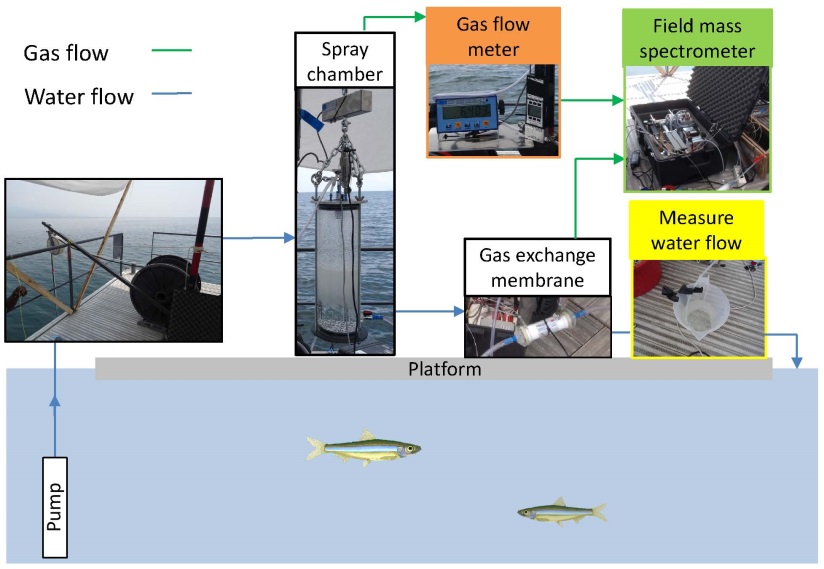


Figure 1: Sketch of measurement approach: lake water is pumped up continuously and dispersed into a spray chamber. The equilibrated gas and water phases flow out of the spray chamber at the top and bottom, respectively. For both phases, partial gas pressures and flows are measured.

A custom-made spray chamber (d = 12 cm, h = 40 cm, V = 4.5 L) is subsequently used to separate water and gas phase of the sampling water by dispersing it through a nozzle at the top. Gas and water phases leave the spray chamber through an outlet at the top and at the bottom, respectively. Pressure (Wika P-30, accuracy: 1 hPa) and temperature (Maxim DS18B20, accuracy: 0.5 °C) are recorded continuously for both gas and water while gas flow is measured using a laminar flow meter (Alicat Scientific MBS-20SLPM-D) and water flow using a simple bucket. Finally, the gas phase components are directly analyzed in the field mass spectrometer. In order to quantify the remaining gas in the sample water, the headspace of a membrane module is equilibrated with the sampling water flowing from the spray chamber and also analyzed using the mass spectrometer.

Table 1: Pump operation data

|  | **Pump** | **Tube** | **Flow** | **Tube flushed** |
| --- | --- | --- | --- | --- |
| Above 250 m | Yes | polyamide, 6 mm | ~ 1.6 L/min | 2x |
| 250 – 310 m | No | polyamide, 10 mm | ~ 0.5 L/min | 2x |
| Below 310 m | No | polyamide, 10 mm | ~ 1 L/min | 2x |

There are two crucial components in the measurement approach: the portable field mass spectrometer, developed at Eawag (Brennwald et al., 2016, see next section) and the gas flow meter MBS-20SLPM-D made by Alicat Scientific. The gas flow meter forces the gas through a laminar flow element and records the pressure drop across the element. This pressure drop is a linear function of gas flow and thus directly allows the instrument to calculate the gas flow for a given gas mixture. The range of the instrument is from 0.1 to 20 l/min and its accuracy depends on the average flow measured (between 2.5 and 8.5 % below 250 m).


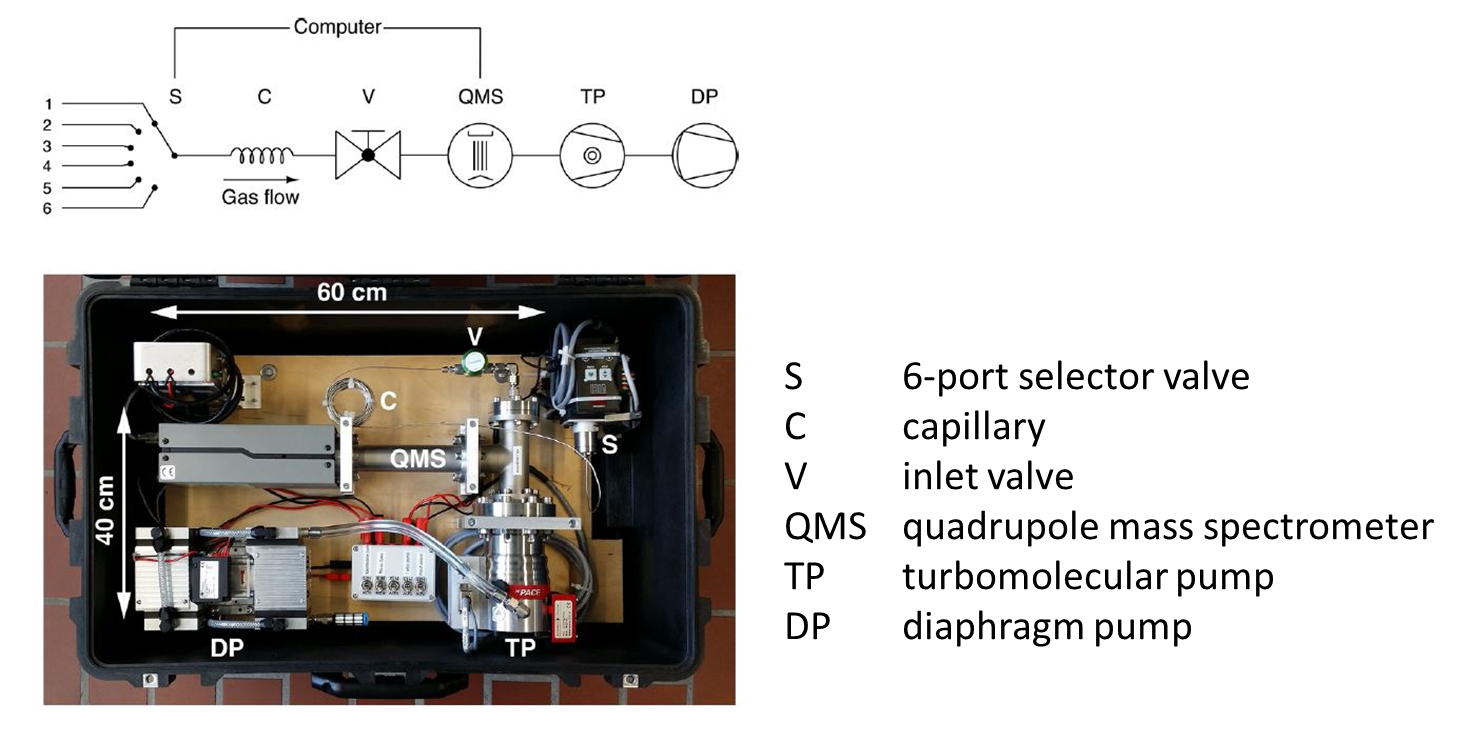


Figure 2: On-site mass spectrometer developed at Eawag (Brennwald et al., 2016)

Figure 2 shows the main components as well as sketch of the mass spectrometric system. Sample or calibration gas enters through one of the ports of the multi-port selector valve and enters the quadrupole mass spectrometer (QMS) through a long capillary. The QMS is evacuated using a low and a high vacuum pump and is suitable for the analysis of any gas between 0 and 200 atomic mass (atm).

If the sample medium is gas (e.g. the gas from deep water in Lake Kivu), the sample can directly enter the QMS. But if water is sampled (down to 130 m in Lake Kivu and for residual gas concentration in water below 130 m), the gas contained in the water needs to be extracted into a headspace. In our approach, this is done using a Liqui-Cel G542 membrane module. Ideally, the water flow through this membrane should be higher than ~ 1 L/min in order to guarantee an equilibrium in the headspace despite the slow consumption by the QMS. Using the equilibrium assumption, the gas concentration in the water can be deduced using the Henry coefficient, and membrane properties do not have to be taken into account.

The device was calibrated several times per day using the following calibration gases (contained in Linde Plastigas bags and permanently attached to two inlets):

- Cal gas 1: 20 % CH_4_, 80 % CO_2_
- Cal gas 2: 30 % CH_4_, 60 % CO_2_, 10 % air

###

### Calculations

In order to compute in-situ concentrations from concentrations in the gas and water phase, we use the following equation:

$C_{in situ}\left[ \frac{mmol}{L} \right]= \frac{Q_{gas}\left[ \frac{L_{gas,SATP}}{min} \right]}{Q_{water}\left[ \frac{L}{min} \right]}C_{gas}\left[ \frac{mmol}{L_{gas, SATP}} \right]+C_{water}\left[ \frac{mmol}{L} \right]$ (1)

where C is concentration and Q is flow. Q_water_ is measured using a bucket and Q_gas_ is the gas flow at standard ambient temperature and pressure SATP (T = 25°C and P = 1013 hPa) computed according to equation (2).

$Q_{gas} \left[ \frac{L_{gas, SATP}}{min} \right]=\frac{V_{out,SATP}+\left( M_{init}-M_{final} \right)1\left[ \frac{L}{kg} \right]\frac{P_{ambient}}{P_{SATP}}}{t}, P_{ambient}=855 hPa$ (2)

V_out,SATP_ is the total outflow volume (in L_gas,SATP_) measured by the gas flow meter during the time t (in min). M_init_ and M_final_ are the weight (kg) of the spray chamber at the start and at the end of a gas flow measurement (usually 20-30 minutes) to take into account the variation of the gas volume in the spray chamber. Finally, the pressure correction term ensures coherences with the output of the gas flow meter in SATP (temperature correction is negligible). P_ambient_ is the ambient pressure at Lake Kivu, which was always close to 855 hPa.

After calibration, the mass spectrometer gives out relative partial pressure values for every major gas component (CH_4_, CO_2_, N_2_ and O_2_). Water vapor is not measured but taken into account assuming saturation and by calculating saturation pressure (atm) according to the following equation (Robinson, 1954):

$\ln(pH_{2}O)=24.4543-67.4509\left( \frac{100}{T} \right)-4.8489\ln\left( \frac{T}{100} \right)-0.000544S$ (3)

where T (°K) is water temperature, and S (‰) is salinity, which is calculated from conductivity and ionic composition (Wüest et al., 1996). Finally, the relative pressures are converted to absolute pressures using pressure gauges at the inlet of the mass spectrometer.

The partial pressures are subsequently converted to concentrations. For the pure gas phase coming out of the spray chamber, this is done using the molar volume at SATP (24.46 L/mol at 25°C and 1013 hPa) in order to get the concentration in$\left[ \frac{mmol}{L_{gas,SATP}} \right]$. For the water phase, we use the Henry coefficients for CH_4_ (Yamamoto, 1976) and CO_2_ (Weiss 1974) which results in concentration in $\left[ \frac{mmol}{L} \right].$

Individual uncertainties

We first assess the accuracy of the individual concentration and flow measurements in order to conclude on the total uncertainty of our approach.

1. Mass spectrometer


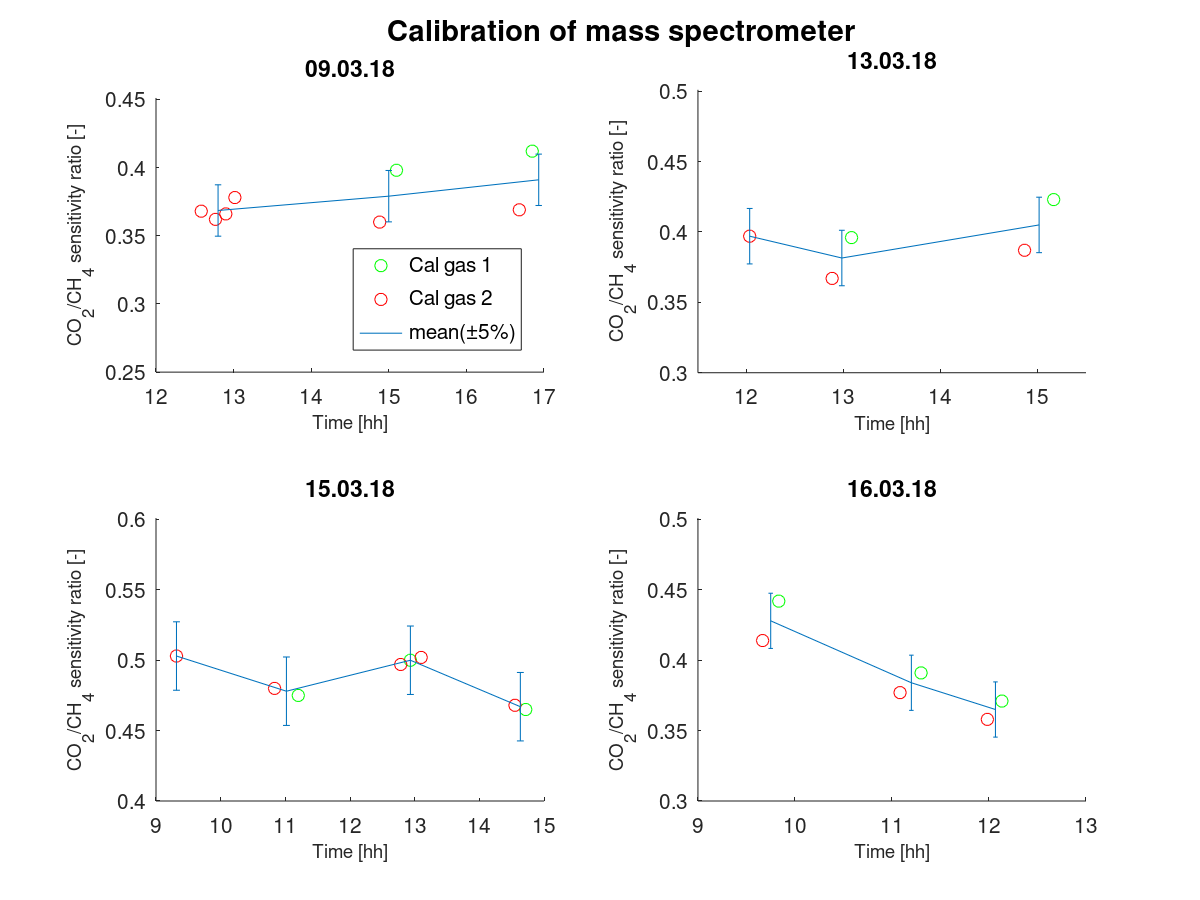


Figure 3: Variability of CO_2_ to CH_4_ sensitivity ratio of two different calibration gases for different measurement days. The sensitivity ratio of a gas is defined as the signal strength in [A] divided by its partial pressure in the calibration gas in [hPa].

Figure S3 depicts the CO_2_/CH_4_ sensitivity ratio for two different calibration gases (see section 2.6.2 for the composition of the gases) over several measurement days. Most deep water samples were taken on these days. The sensitivity of a gas is given by the detector signal in Ampère divided by its partial pressure in the calibration gas mixture [A/hPa]. On 9, 13 and 16 March, the sensitivity ratio was significantly higher for calibration gas 1 than for calibration gas 2. However, no clear indication was found to discard any of the two calibration gases and therefore both gases were used for calibration purposes. The corresponding uncertainty range of the sensitivity ratio was estimated to 10 % resulting in a deviation of ± 5 % from the mean value (blue error bars in Figure S3).

In the following, the individual uncertainties of the CH_4_ and CO_2_ measurements are estimated from the uncertainty of the sensitivity ratio

*E(CO_2__sens/CH_4__sens) = ± 5 %*

where E(…) means uncertainty.

The sensitivities are obtained by dividing the calibration signal [A] by the partial pressure in the calibration gas [hPa]. The calibration signals for both CO_2_ and CH_4_ (and their uncertainties) are not related to each other. Thus, assuming that CH_4_ and CO_2_ have the same relative uncertainty, we get:

*E(CO_2__sens) = E(CH_4__sens) = ± 2.5 %*

(from uncertainty propagation of the ratio of two independent variables for small uncertainty).

To obtain the raw result of a sample, CH_4__sens is multiplied by “CH_4__signal” which is the signal measured at a certain depth in [A]. This raw result is called “CH_4__meas”. The uncertainty of CH_4__signal due to the variability of the individual mass peak measurements is *± 1 %*. Therefore:

*E(CH_4__meas) = E(CH4_sens) + E(CH_4__signal) = 2.5 % + 1 % = ± 3.5 %.*

The same is valid for CO_2__meas.

The final result consists of the measured quantity normalized by the ratio of total pressure (uncertainty around 0.1 % which is neglected here) and total gas pressure found by the mass spectrometer. Total gas pressure of the mass spectrometer is very close to CO_2__meas + CH_4__meas below 250 m and therefore: *E(CH_4__result) = E(CH_4__meas/(CH_4__meas + CO_2__meas))*

*E(CO_2__result) = E(CO_2__meas/(CH_4__meas + CO_2__meas))*

The denominator of these equations not only depends on the error of both CH_4__meas and CO_2__meas but also on the ratio of these measured values. Moreover, the nominator and denominator are not independent, thus making a general assessment difficult. Thus, we proceed with the calculation of “extreme” cases (all errors add up) and we find for a typical CH_4_/CO_2_ ratio of 0.2/0.8 = 0.25:

E(CH_4__result) ≅ ± 5.5 %

E(CO_2__result) ≅ ± 1.5 %

The ratio of these errors is close to 4 because at the given composition of the gas phase, a 1 % increase in CO_2_ concentration roughly results in a 4 % decrease in CH_4_ concentration.

Note that in Table 2, these uncertainties were calculated using measured CH_4_/CO_2_ ratios and not the “typical” one.

1. Henry coefficient

In the case of CO_2_, a large fraction of the gas remains dissolved in the sample water. For determining its concentration, the measured partial pressure is multiplied by the temperature-dependent Henry coefficient. We adopt a total uncertainty of 3 % to account for the uncertainty of the temperature-dependence of the Henry coefficient and for the fact that the equilibrium in the membrane contactor might not be perfect.

For CH_4_, the uncertainty in the Henry coefficient can be neglected, since only a small fraction of the gas remains in the water after degassing.

1. Gas flow

The gas flow is computed using equation (2). According to the manufacturer, the accuracy of the total gas volume V_out,SATP_ which passed through the gas meter in time t is given by

$\Delta_{V_{out,SATP}}\left[ \% \right]=\frac{0.8\%\frac{V_{out,SATP}}{t}+0.2\%Q_{Full scale}}{\frac{V_{out,SATP}}{t}} , Q_{Full scale}=20 L/min$ (4)

With Q_Full scale_ the highest gas flow measurable by the device. This error is between 2.5 and 8.5 % in the deep water due to the high full scale value of the gas meter. Thus the contributions of the hanging scales and the pressure gauge (both 0.1 % accuracy) are negligible in comparison and we can state

$\Delta_{Q_{gas}}=\Delta_{V_{out,SATP}}$ = 2.5 – 8.5 % (5)

1. Water flow

We used a 5 L bucket with labelling every 50 mL, usually filled to 3 to 4 L. Experiments in the laboratory show a positive bias of 3 % and random fluctuations of ± 1 % (Figure 4). The positive bias was corrected by multiplying the measured water flow by 0.97. We estimated the accuracy of the corrected water flow to be ± 1 %.


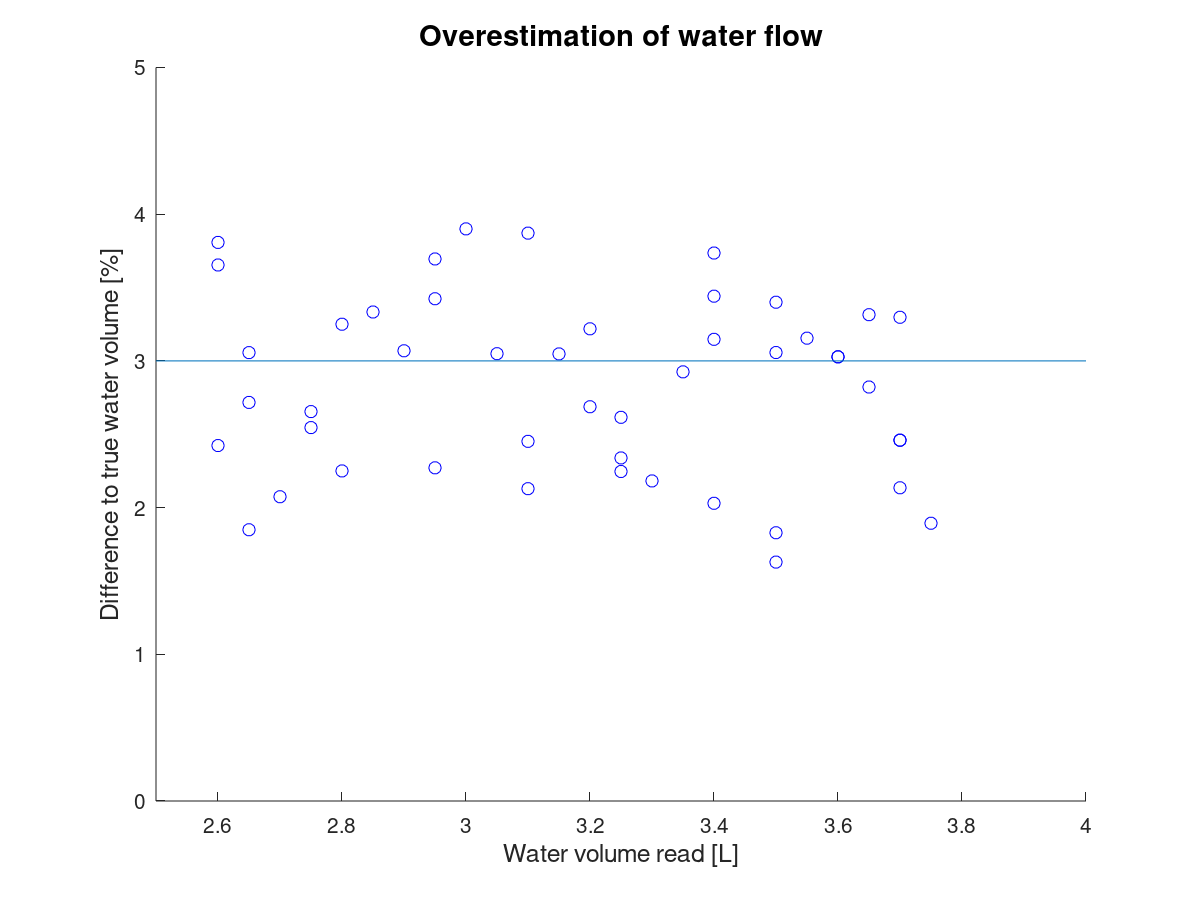


Figure 4: Bias and uncertainty of water flow measurement. A laboratory scale was used to estimate the true water volume.

Total accuracy

We estimated the individual accuracies above, so the true values of the individual measurements (Q_gas_, Q_water_ and MS measurement) should lie within those limits. In the worst case, we will either underestimate or overestimate the true value of all those individual measurements at the same time. Assuming that all individual errors are small, the maximum uncertainty of the gas amount contained only in the gas phase is given by the sum of the relative errors. We give here an example for the calculation of the uncertainty of CH_4_ and CO_2_ at 410 m depth.

CH_4_:

$\Delta_{{CH}_{4}}=\Delta_{Q_{gas}}+\Delta_{Q_{water}}+\Delta_{Conc.}=9.2 \%$ (6)

$$\mathrm{with}\Delta_{Q_{gas}}=2.7 \%, \Delta_{Q_{water}}=1 \%, \Delta_{Conc.}=5.5 \%$$

This means that in the worst case, the true value in the gas phase is under- or overestimated by around 9 %. The error contribution from the CH_4_ remaining in the water phase is neglected as it is around 1 % of the total CH_4_.

CO_2_:

The uncertainty of the CO_2_ measurement is a weighted sum of the uncertainties in the gas and water phase respectively

$\Delta_{{{CO}_{2}}_{gas}}=\Delta_{Q_{gas}}+\Delta_{Q_{water}}+\Delta_{Conc.}=5.2 \%$ (7)

$$\mathrm{with}\Delta_{Q_{gas}}=2.7 \%, \Delta_{Q_{water}}=1 \%, \Delta_{Conc.}=1.5 \%$$

$\Delta_{{{CO}_{2}}_{water}}=\Delta_{Conc.}+ \Delta_{\mathrm{Henry}}=4.5 \%$ (8)

$\mathrm{with}\Delta_{Conc.}=1.5 \%, \Delta_{Henry}=3 \%$

Therefore, the accuracy of CO_2_ is the weighted average of equations (7) and (8), closer to equation (7) in the deep water and almost equal to equation (8) at the lake surface.

In general, the uncertainty of CH_4_ is around 10 % and around 5 % for CO_2_ in the deep water (below 250 m).

References:

Brennwald, M. S., Schmidt, M., Oser, J., and Kipfer, R. (2016). A portable and autonomous mass spectrometric system for on-site environmental gas analysis. *Environmental Science & Technology*, *50*(24), 13455-13463.

Moore, J. C., Battino, R., Rettich, T. R., Handa, Y. P., and Wilhelm, E. (1982). Partial molar volumbes of gases at infinite dilution in water at 298.15 K. *Journal of Chemical and Engineering Data*, *27*(1), 22-24.

Rettich, T. R., Handa, Y. P., Battino, R., and Wilhelm, E. (1981). Solubility of gases in liquids. 13. High-precision determination of Henry's constants for methane and ethane in liquid water at 275 to 328 K. *The Journal of Physical Chemistry*, *85*(22), 3230-3237.

Robinson R. A. (1954). The vapour pressure and osmotic equivalence of sea water. *Journal of the Marine Biological* Association *of the* United Kingdom 33(2): 449-455.

Ross, K. A., Gashugi, E., Gafasi, A., Wüest, A., & Schmid, M. (2015). Characterisation of the subaquatic groundwater discharge that maintains the permanent stratification within Lake Kivu; East Africa. *PloS one*, *10*(3), e0121217.

Weiss, R. (1974). Carbon dioxide in water and seawater: the solubility of a non-ideal gas. *Marine chemistry*, *2*(3), 203-215.

Wüest, A., G. Piepke, and J. D. Halfman (1996), Combined effects of dissolved solids and temperature on the density stratification of Lake Malawi, in *The Limnology, Climatology and Paleoclimatology of the East African Lakes*, edited by T. C. Johnson and E. O. Odada, pp. 183–202, Gordon and Breach, New York.

Yamamoto, S., Alcauskas, J. B., and Crozier, T. E. (1976). Solubility of methane in distilled water and seawater. *Journal of Chemical and Engineering Data*, *21*(1), 78-80.
