# Supplementary material for: No increasing risk of a limnic eruption at Lake Kivu: Intercomparison study reveals gas concentrations close to steady state
Source: PLoS One. 2020 Aug 25;15(8):e0237836. doi: 10.1371/journal.pone.0237836 (PMC7446963; doi:10.1371/journal.pone.0237836)
Supplement: S2 Appendix — (DOCX) [file pone.0237836.s005.docx]

**No increasing risk of a limnic eruption at Lake Kivu: intercomparison study reveals gas concentrations close to steady state**

Fabian Bärenbold^1^*, Bertram Boehrer^2^, Roberto Grilli^3^, Ange Mugisha^4^, Wolf von Tümpling^2^, Augusta Umutoni^4^, Martin Schmid^1^

**S2 Appendix. Calculation of salinity effect of Lake Kivu dissolved solids**

The dependence on salinity of the Henry coefficient has been derived for sea salt. However, the dissolved salts in Lake Kivu mainly consist of bicarbonates of Na, Mg, K, and Ca. We assume that the salinity effect mainly depends on the ionic strength of the solution. For seawater, the ionic strength I (mol/kg) can be calculated from unit less (i.e., kg/kg) salinity as $I=C_{2}*\frac{S}{1-S}$ (IOC et al., 2010) with C_2_ = 19.8272 mol/kg. Since both the salinity correction of the Henry coefficient and the ionic strength in Lake Kivu are small, we can neglect the denominator and replace S in equation (2) by 1000 g/kg * I/C_2_. The ionic strength in Lake Kivu was calculated as a function of conductivity, based on the observed average concentrations of the main dissolved ions and conductivities given in Tables 2 and 3 of Ross et al. (2015). A linear regression forced through the zero point resulted in an equation of I = C_3_ κ_25_ (R^2^ = 0.985), where κ_25_ (mS/cm) is the conductivity corrected to a standard temperature of 25 °C, and C_3_ = 0.0173 (mol/kg)/(mS/cm). In summary, this results in the following equation for the Henry coefficients of CO_2_, CH_4_ and N_2_ in Lake Kivu:

$$ln\left( K_{i} \right)=A_{1}+A_{2}\left( 100/T \right)+A_{3}\ln\left( T/100 \right)+C_{1}\kappa_{25}\left[ B_{1}+B_{2}\left( T/100 \right)+B_{3}\left( T/100 \right)^{2} \right]$$

where C_1_ = 1000*C_3_ /C_2_ = 0.8725 (g/kg)/(mS/cm). Altogether, water with a conductivity of 1 mS/cm in Lake Kivu has about the same ionic strength as seawater with a salinity of 0.8725 g/kg. Finally, it should be noted that for CO_2_, this Henry coefficient is the ratio between the fugacity of CO_2_ and the concentration of the dissolved undissociated aqueous CO_2_, often referred to as H_2_CO_3_ . For calculating the total dissolved CO_2_, the concentration of bicarbonate (HCO_3_^-^) and carbonate (CO_3_^2-^) would have to be added.
