# Supplementary material for: No increasing risk of a limnic eruption at Lake Kivu: Intercomparison study reveals gas concentrations close to steady state
Source: PLoS One. 2020 Aug 25;15(8):e0237836. doi: 10.1371/journal.pone.0237836 (PMC7446963; doi:10.1371/journal.pone.0237836)
Supplement: S3 Appendix — (DOCX) [file pone.0237836.s006.docx]

**No increasing risk of a limnic eruption at Lake Kivu: intercomparison study reveals gas concentrations close to steady state**

Fabian Bärenbold^1^*, Bertram Boehrer^2^, Roberto Grilli^3^, Ange Mugisha^4^, Wolf von Tümpling^2^, Augusta Umutoni^4^, Martin Schmid^1^

**S3 Appendix. Octave scripts for conversion of concentration to partial pressure**

- The script “press2conc.m” computes partial pressures and total dissolved gas pressure from concentration profiles.
- “Compute_fugacity.m” calculates the fugacity coefficients (based on Ziabakhsh-Ganji and Kooi 2012)
- Solubility for CH_4_, CO_2_ and N_2_ are computed in functions according to Wiesenburg and Guinasso 1979, Weiss 1974 and Weiss 1970.

%%% Octave script to convert CH4, CO2 and N2 concentrations in Lake Kivu to pressure

%%% Written by Martin Schmid and Fabian Bärenbold, Eawag, 2019

clear all

close all

% load gas profiles

load C:\Users\username\your_gas_data.mat

% Extract data

depth = depth_gas;

CH4 = conc_CH4;

CO2 = conc_CO2;

N2 = conc_N2;

% Compute pressure in bar (including atmospheric pressure)

p = depth*0.978/10 + 0.855;

% Load conductivity and temperature profiles

load C:\Users\username\your_CTD_data.mat

% Extract data

depth2 = depth_CTD;

k25 = k25_CTD;

Tin = T_CTD;

% Interpolate conductivity and temperature on the same grid as gases

C = interp1(depth2,k25_CTD,depth);

T = interp1(depth2,T_CTD,depth);

% Salinity as a function of conductivity (see SI of this publication, Bärenbold et al., 2019)

S = 0.8725 * C;

% Initialize arrays for fugacity and solubility

phi_CH4 = zeros(size(depth));

phi_CO2 = zeros(size(depth));

phi_N2 = zeros(size(depth));

KH_CH4 = zeros(size(depth));

KH_CO2 = zeros(size(depth));

KH_N2 = zeros(size(depth));

% Initialize mixing ratios

CH4_mix = 0.18*ones(size(depth));

CO2_mix = 0.80*ones(size(depth));

N2_mix = 0.02*ones(size(depth));

% Initialize counter

k = 0;

% Iterate while CH4_mix has not converged

do

CH4_mix_old = CH4_mix;

CO2_mix_old = CO2_mix;

N2_mix_old = N2_mix;

% Calculate fugacities and solubilities with current mixing ratios

for i = 1:length(depth)

[phi_CO2(i) phi_CH4(i) phi_N2(i)] = compute_fugacity(CO2_mix_old(i),CH4_mix_old(i), N2_mix_old(i),p(i),T(i));

KH_CH4(i) = Solubility_CH4_Wiesenburg(T(i),C(i),p(i));

KH_CO2(i) = Solubility_CO2_Weiss(T(i),C(i),p(i));

KH_N2(i) = Solubility_N2_Weiss(T(i),C(i),p(i));

end

% Compute new partial pressure and mixing ratios

p_CH4_calc = CH4./KH_CH4./phi_CH4;

p_CO2_calc = CO2./KH_CO2./phi_CO2;

p_N2_calc = N2./KH_N2./phi_N2;

CO2_mix = p_CO2_calc./(p_CO2_calc + p_CH4_calc + p_N2_calc);

CH4_mix = p_CH4_calc./(p_CO2_calc + p_CH4_calc + p_N2_calc);

N2_mix = p_N2_calc./(p_CO2_calc + p_CH4_calc + p_N2_calc);

% Show counter

k = k + 1

% Until convergence

until(abs(CH4_mix - CH4_mix_old)<1e-6)

% Compute total pressure

p_tot = p_CH4_calc + p_CO2_calc + p_N2_calc;

function [phi_CO2 phi_CH4 phi_N2] = compute_fugacity (Z_CO2, Z_CH4, Z_N2, P, T)

% Function to calculate fugacity according to Ziabakhsh-Ganji and Kooi, 2012

% Original Maple Script provided by Zaman Ziabakhsh-Ganji

% transcribed to Octave by Martin Schmid, September 2018

%Indices

%1:CO2

%2:CH4

%3:N2

% Input arguments: Gas mixing ratios, pressure (bar), temperature (C)

T = T + 273.15;

% Acentric factors, critical temperatures and pressures

omega(1)=0.228; Tc(1)=304.25; Pc(1)=73.866;

omega(2)=0.0115; Tc(2)=190.45; Pc(2)=45.96;

omega(3)=0.04; Tc(3)=126.15; Pc(3)=33.999;

% Mole fractions of dissolved gases

Z(1)=Z_CO2;

Z(2)=Z_CH4;

Z(3)=Z_N2;

% Gas constant

R=83.1447;

B = 19654.320 + 147.37*(T - 273.15) - 2.21554*(T - 273.15)^2 + 1.0478e-2*(T - 273.15)^3 - 2.2789e-5*(T - 273.15)^4;

alpha(1) = (1 + (0.37464 + 1.5422*omega(1) - 0.26992*omega(1)^2)*(1 - (T/Tc(1))^0.5))^2;

alpha(2) = (1 + (0.37464 + 1.5422*omega(2) - 0.26992*omega(2)^2)*(1 - (T/Tc(2))^0.5))^2;

alpha(3) = (1 + (0.37464 + 1.5422*omega(3) - 0.26992*omega(3)^2)*(1 - (T/Tc(3))^0.5))^2;

a(1)=(0.45724*R^2*Tc(1)^2*alpha(1))/Pc(1);

b(1)=0.07780*R*Tc(1)/Pc(1);

a(2) = (0.45724*R^2*Tc(2)^2*alpha(2))/Pc(2);

b(2) = 0.07780*R*Tc(2)/Pc(2);

a(3) = (0.45724*R^2*Tc(3)^2*alpha(3))/Pc(3);

b(3) = 0.07780*R*Tc(3)/Pc(3);

B(1) = (b(1)*P)/(R*T);

B(2) = (b(2)*P)/(R*T);

B(3) = (b(3)*P)/(R*T);

K_N2CO2 = 0.2187;

K_CH4CO2 = 0.3349;

a_N2CO2 = (1 - K_N2CO2)*(a(1)*a(3))^(0.5);

a_CH4CO2 = (1 - K_CH4CO2)*(a(1)*a(2))^(0.5);

a_m = Z(1)^2*a(1) + Z(2)^2*a(2) + 2*Z(1)*Z(2)*(a(1)*a(2))^0.5*(1 - K_CH4CO2);

b_m = b(3)*Z(3) + b(1)*Z(1) + b(2)*Z(2);

Am = (a_m*P)/(R*T)^2;

Bm = (b_m*P)/(R*T);

z = roots ([1, -(1-Bm), (Am - 2*Bm - 3*Bm^2), -(Am*Bm-Bm^2 - Bm^3)]);

z = z(imag(z)==0);

Zh = max(z);

Zl = min(z);

G = ((Zh - Zl) + log((Zl - Bm)/(Zh - Bm)) - Am/(Bm*(-2*1.414213562))* ...

log((Zl + (1 + sqrt(2))*Bm)/(Zl + (1 - sqrt(2))*Bm)*(Zh + (1 - sqrt(2))*Bm)/(Zh + (1 + sqrt(2))*Bm)));

if (real(G) < 0)

z = max(z);

else

z = min(z);

end

% Fugacities

phi(1)=exp((B(1)/Bm)*(z - 1) - log(z-Bm) + (Am/(2.828*Bm))*((B(1)/Bm) - (2/a_m)*(Z(1)*a(1) + ...

Z(3)*a_N2CO2 + Z(2)*a_CH4CO2))*log((z + 2.414*Bm)/(z - 0.414*Bm)));

phi(2)=exp((B(2)/Bm)*(z - 1) - log(z - Bm) + (Am/(2.828*Bm))*((B(2)/Bm) - (2/a_m)*(Z(2)*a(2) + ...

Z(1)*a_CH4CO2))*log((z + 2.414*Bm)/(z - 0.414*Bm)));

phi(3)=exp((B(3)/Bm)*(z - 1)-log(z - Bm) + (Am/(2.828*Bm))*((B(3)/Bm) - (2/a_m)*(Z(3)*a(3) + ...

Z(1)*a_N2CO2))*log((z + 2.414*Bm)/(z - 0.414*Bm)));

phi_CO2 = phi(1);

phi_CH4 = phi(2);

phi_N2 = phi(3);

function K_CH4 = Solubility_CH4_Wiesenburg (T,C,P)

% Martin Schmid, Eawag, September 2018

% Input: Temperature T in °C, Specific Conductivity C in ms/cm, Pressure P

% in bar.

% Calculates the solubility of CH4 expressed in mol/L/atm following the

% equations of Wiesenburg and Guinasso (1979) for the case of Lake Kivu.

% Note that the original publication calculates dimensionless

% Bunsen coefficients, therefore the first parameter in the equation

% is different. Also, in the original publication salinity is defined for sea

% salt. Here we use conductivity in Lake Kivu instead and calculated at

% which conductivity the water of Lake Kivu (based on it's main salt

% composition) has the same ionic strength as sea water with a certain

% salinity

%

% Absolute temperature in °K

T_abs = 273.15 + T;

% Gas constant in J/K/mol

R = 8.31446;

% Lake Kivu salinity equivalent

S = 0.8752*C;

% Dimensionless Bunsen solubility according to Wiesenburg and Guinasso, 1979

K_CH4 = exp(-68.8862 + 101.4956*(100/T_abs) + 28.7314*log(T_abs/100) + ...

S*(-0.076146 + 0.043970*(T_abs/100) - 0.006872*(T_abs/100)^2));

% Transformation to mmol/l/bar

K_CH4 = K_CH4/R/273.15*1e5;

% Partial molar volume in cm3/mol, Rettich et al. (1981)

v_CH4 = exp(3.541 + 0.00123*T);

% Pressure correction according to Weiss 1974

p_corr = exp((1.01325 - P)*v_CH4/10/R/T_abs);

% Solubility corrected for pressure

K_CH4 = p_corr*K_CH4;

function K_CO2 = Solubility_CO2_Weiss (T,C,P)

% Martin Schmid, Eawag, September 2018

% Input: Temperature T in °C, Specific Conductivity C in ms/cm, Pressure P

% in bar.

% Calculates the solubility of CO2 expressed in mol/L/atm following the

% equations of Weiss (1974) for the case of Lake Kivu.

% In the original publication salinity is defined for sea

% salt. Here we use conductivity in Lake Kivu instead and calculated at

% which conductivity the water of Lake Kivu (based on it's main salt

% composition) has the same ionic strength as sea water with a certain

% salinity

%

% Absolute temperature in °K

T_abs = 273.15 + T;

% Gas constant in J/K/mol

R = 8.31446;

% Lake Kivu salinity equivalent

S = 0.8752*C;

% Solubility in mol/l/atm according to Weiss 1974

K_CO2 = exp(-58.0931 + 90.5069*(100/T_abs) + 22.2940*log(T_abs/100) + ...

S*(0.027766 - 0.0258880*(T_abs/100) + 0.0050578*(T_abs/100)^2));

% Transformation to mmol/l/bar

K_CO2 = K_CO2*0.986923*1000;

% Molar volume in cm3/mol, Weiss 1974

v_CO2 = 32.3;

% Pressure correction according to Weiss 1974

p_corr = exp((1.01325 - P)*v_CO2/10/R/T_abs);

% Solubility corrected for pressure

K_CO2 = p_corr*K_CO2;

function K_N2 = Solubility_N2_Weiss (T,C,P)

% Fabian Bärenbold, Eawag, May 2019

% Input: Temperature T in °C, Specific Conductivity C in ms/cm, Pressure P

% in bar.

% Calculates the solubility of N2 expressed in mol/L/atm following the

% equations of Weiss (1970) for the case of Lake Kivu.

% In the original publication salinity is defined for sea

% salt. Here we use conductivity in Lake Kivu instead and calculated at

% which conductivity the water of Lake Kivu (based on it's main salt

% composition) has the same ionic strength as sea water with a certain

% salinity

%

% Absolute temperature in °K

T_abs = 273.15 + T;

% Gas constant in J/K/mol

R = 8.31446;

% Lake Kivu salinity equivalent

S = 0.8752*C;

% Dimensionless Bunsen solubility according to Weiss 1970

K_N2 = exp(-59.6274 + 85.7661*(100/T_abs) + 24.3696*log(T_abs/100) + ...

S*(-0.051580 + 0.026329*(T_abs/100) - 0.0037252*(T_abs/100)^2));

% Conversion of Bunsen to Henry coefficient in [mmol/l/bar]

K_N2 = K_N2/R/273.15*1e5;

% Partial molar volume in cm3/mol at 25°C, Moore et al. 1982

v_N2 = 35.7;

% Pressure correction according to Weiss 1974

p_corr = exp((1.01325 - P)*v_N2/10/R/T_abs);

% Solubility corrected for pressure

K_N2 = p_corr*K_N2;

References:

Weiss, R. F. (1970). The solubility of nitrogen, oxygen and argon in water and seawater. In *Deep Sea Research and Oceanographic Abstracts* (Vol. 17, No. 4, pp. 721-735). Elsevier.

Weiss, R. (1974). Carbon dioxide in water and seawater: the solubility of a non-ideal gas. *Marine chemistry*, *2*(3), 203-215.

Wiesenburg, D. A., and Guinasso Jr, N. L. (1979). Equilibrium solubilities of methane, carbon monoxide, and hydrogen in water and sea water. *Journal of chemical and engineering data*, *24*(4), 356-360.

Ziabakhsh-Ganji, Z., and Kooi, H. (2012). An Equation of State for thermodynamic equilibrium of gas mixtures and brines to allow simulation of the effects of impurities in subsurface CO2 storage. *International Journal of Greenhouse Gas Control*, *11*, S21-S34.
